# The Study of Short-Term Plastic Visual Perceptual Training Based on Virtual and Augmented Reality Technology in Amblyopia

**DOI:** 10.1155/2022/2826724

**Published:** 2022-09-01

**Authors:** Fan Tan, Xubo Yang, Yuchen Fan, Yongchuan Liao

**Affiliations:** ^1^Department of Ophthalmology, West China Hospital, Sichuan University, Chengdu, China; ^2^Department of Ophthalmology, West China-Guang'an Hospital, Sichuan University, Guang'an, Sichuan, China

## Abstract

**Backgrounds:**

The treatment for amblyopia can have a substantial impact on quality of life. Conventional treatments for amblyopia have some limitations, then we try to explore a new and effective method to treat amblyopia. This study aimed to determine the potential effect of short-term plastic visual perceptual training based on VR and AR platforms in amblyopic patients.

**Methods:**

All observers were blinded to patient groupings. A total of 145 amblyopic children were randomly assigned into 2 groups: VR group (71 patients) and AR group (74 patients). In the VR group, each subject underwent a 20-min short-term plastic visual perceptual training based on a VR platform, and in the AR group, based on an AR platform. The best-corrected visual acuity (BCVA), fine stereopsis, and contrast sensitivity function (CSF) were measured before and after training.

**Results:**

The BCVA (*P* < 0.001) and fine stereopsis (*P* < 0.05) were improved significantly both in VR and AR group after training. Moreover, in the AR group, the CSF showed the value of all spatial frequencies had a statistically signiﬁcant improvement after training (*P* < 0.05), while in the VR group, only the value of spatial frequency 12 improved significantly (*P* = 0.008).

**Conclusions:**

This study showed that the short-term plastic visual perceptual training based on VR and AR technology can improve BCVA, fine stereopsis and CSF of refractive amblyopia. It was suggested that the visual perceptual training based on the VR and AR platforms may be potentially applied in treatment for amblyopia and provided a high-immersing alternative.

## 1. Introduction

Amblyopia is a vision function developmental disorder, during a critical period of vision ontogeny [1–3]. The typical symptom of amblyopia is characterized as reduced best-corrected visual acuity (BCVA) in one or both eyes [4]. Besides reduced visual acuity, amblyopia also causes the damage of visual function, like contrast sensitivity function (CSF) [5], interocular suppression (IS) [6], foveal crowding [7], and stereopsis [2]. Conventional treatments for amblyopia rely on the forced use of the amblyopic eye by depriving the vision of the healthy fellow eye (by patching or penalizing). [1] Patching, a 200-year long amblyopia treatment, has the compliance decrease in therapy. Initially, most children can just complete 60% of the clinician-recommended daily dose (in hours per day) of patching therapy. Even worse, they can complete only 40% after 60 days [8]. The most common method of penalization is to use atropine mydriasis to penalize the healthy eye, which does not affect the appearance, then has a better compliance than patching. However, atropine may have some side effects such as fever, skin flush, dysphoria, and glare. Moreover, both patching and penalization may destroy the binocular vision condition and damage the binocular vision function [9].

Virtual reality (VR), the presentation of computer-generated 3D environments, enables users to become fully immersed in a simulated world in which they can interact via multiple sensory channels: visual, auditory, or haptic. [10] Different from VR, augmented reality (AR) is a live direct view of a physical, real-world environment whose elements are augmented by computer-generated sensory input such as sound, video, and graphic data. [11, 12] Thus, AR also exhibits high immersion and interaction. Furthermore, users can see the real-world environment and the computer-generated objects simultaneously.

VR and AR have been explored in many aspects in ophthalmologic and optometric fields, such as low vision [13], glaucoma [14, 15], strabismus [16, 17], testing binocular imbalance [18, 19], age-related macular degeneration [20], and distance-based vision aid for blind [13]. Several studies aimed to train amblyopia based on VR technology and showed various improvement in visual acuity. [10–12, 21, 22] Comparatively, the study of amblyopia training based on AR technology was rare. Bao et al. focused on long-term potentiation (LTP) of the amblyopia treatment in which the patients adapted to patchwork video images of daily-life environment based on AR technique for several days and showed the improvement of visual function in amblyopic eyes. [22].

Here, in order to explore the potential effect of visual perceptual training based on VR and AR technology in amblyopic treatment, we conduct a pilot study of short-term visual perceptual training based on VR and AR platforms in refractive amblyopic children.

## 2. Materials and Methods

### 2.1. Participants

A total of 145 children who had refractive amblyopia (diagnosed by a Pediatric Ophthalmology Specialist (Y.L.)) were recruited from the ophthalmology department of West China Hospital, Sichuan University, Chengdu, China, from April 24, 2020, to November 24, 2020. This study followed the provisions of the Declaration of Helsinki and was approved by the Ethics Committee of Sichuan University, including the screening, inspection, and data collection. The diagnosed standard was defined as follows: unilateral amblyopia in preschool age children is defined as an interocular difference of greater than or equal to two lines of BCVA, whereas bilateral amblyopia is defined as BCVA in either eye less than 20/40, in children aged 3 to 5 years and less than 20/33 in children aged 6, or older [23]. All subjects with any other ocular disease were excluded in our study.

### 2.2. Trial Protocol

All participants were randomly assigned via the random number table method with equal probability to 2 groups before they received the training. VR group: Each subject underwent a 20-min short-term visual perceptual training based on the VR platform and the AR group underwent a training based on the AR platform. All observers were blinded to patient groupings.

All participants underwent a baseline ocular examination by technical professional workers, including assessments of BCVA, anterior segment examination with slit lamp, fundoscopy, cover test, fine stereoacuity and CSF testing. BCVA, fine stereoacuity, and CFS were measured before and after training. BCVA was measured with the standard E chart at 5 meters. CSF was measured with the functional acuity contrast test (OPTEC® 6500 visual function tester). Fine stereoacuity was measured with fine random dot stereoscopic inspection which was developed by Guangzhou Medical Instrument Research Institute (Guangzhou, China).

## 3. Methods of Short-Term Visual Perceptual Training Based on VR and AR

**Figure 1:**
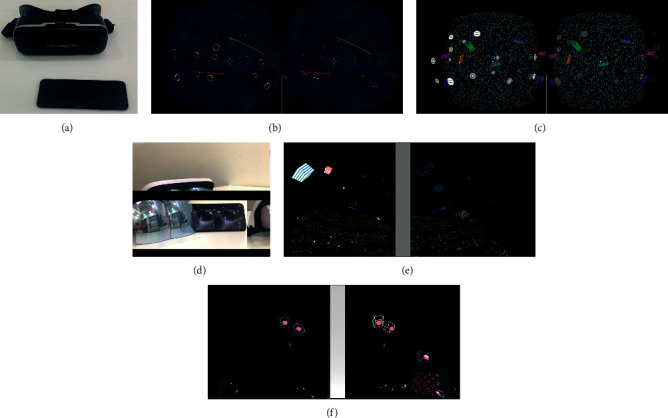
The exhibition of visual perceptual training. (a) The apparatus of VR. (b) The view of Game 1 for VR. (c) The view of Game 2 for VR. (d) The apparatus of AR. (e) The view of Game 1 for AR. (f) The view of Game 2 for AR.

Visual perceptual training was performed by using the beta version of the computer game Diplopia Game (National Engineering Research Center for Healthcare Devices, GuangDong, China). In the VR group, the game was run in a simple virtual reality helmet with a smart phone (Figure 1(a)). In the AR group, the game was run in a simple augmented reality helmet with a smart phone (Figure 1(d)). Each short-term training consists of two different 6–8 min games between which patients have a 5-min break. The two games in the short-term training were differently designed for the AR group and VR group, respectively. The smart phone was equipped with an IPS display (6,1″ diagonal, resolution of 896 × 828 pixels per eye), with a 90° field of view, mounted with accelerometer, gyroscope, and magnetometer sensor for positional tracking system. The smart phones used a Android mobile system.

In the VR group, under dichoptic viewing conditions, some objects are seen with the better eye and others are seen with the amblyopic eye, and the game forces the brain to use both eyes together to play. Game 1 is the cross and circle game. In this game, the circle was visible to the better eye, and the cross was only visible to the amblyopic eye. Patients wear an VR helmet and move head to control the cross to move into the circle, then the circle and cross burst (Figure 1(b)). Game 2 is the Gabor patch shooting game. In this game, the contrast of the Gabor patch model stimulus visible to the good eye was low, while the contrast of the same model stimulus visible to the poor eye was high. Patients put on the VR helmet and move head to control the bullet to shoot the Gabor patch in the state of binocular integration, and then the Gabor patch bursts (Figure 1(c)). During the training process, the signal of better eye can be decreased and the signal of amblyopic eye can be increased (flickers and jitters).

In the AR group, Game 1 is also under dichoptic viewing conditions, but some moving 3D squares appear in the peripheral visual field randomly, and some shooting bullets appear in the central visual field automatically. The signal of better eye is filtered by the Gaussian blur, and the intensity of filtering can be appropriately adjusted, while the signal of amblyopic eye remains unchanged. Patients wear an AR helmet and move head to control the shooting bullet to break the moving 3D squares appearing in the visual field (Figure 1(e)). Game 2 is under the condition of binocular vision and a group of separated balls and cube boxes appeared randomly in the visual field. The balls can only be visible to the amblyopic eye, and the cube boxes can only be visible to the better eye. Patients put on the AR helmet and move head to let the ball enter the cube boxes, then repeat the above task until all balls move into cube boxes. In the training process, the signal of the balls can flicker and shake (Figure 1(f)). During the training process, besides the training game, patients with AR helmet also can see the real-life information at the same time.

### 3.1. Statistical Analysis

All data were expressed as the mean ± standard deviation. Comparisons between the two independent groups were made by using a two-tailed paired samples *t* test. Chi-squared test was used to test for differences in sex. All data were analyzed with SPSS software (version 23.0; SPSS Inc., Chicago, Illinois, USA). *P* values < 0.05 were considered statistically significant.

## 4. Results

### 4.1. Demographics of Patients

A total of 145 refractive amblyopic children were randomly assigned into 2 groups. There were no statistically significant differences in gender (*P* = 0.162), age (*P* = 0.178), or BCVA (*P* = 0.665) of the patients between the 2 groups (Table 1).

### 4.2. Comparison of Visual Function between Pre- And Post-Training

**Figure 2:**
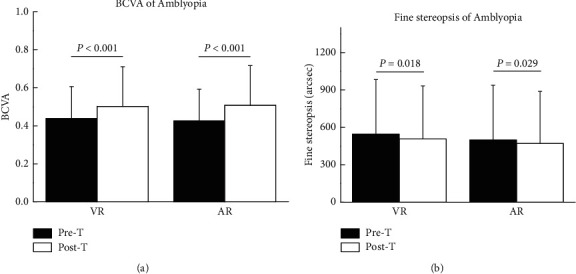
Comparison the BCVA and fine stereopsis between pre- and post-training.

Table 2 summarizes the main clinical data of patients included in this study. As shown, in both of the 2 groups, BCVA improved significantly (*P* < 0.001) (Figure 2(a)).

The comparison of the fine stereopsis between pre- and post-training also showed a significant change in both of the 2 groups (*P* < 0.05) (Table 2, Figure 2(b)).

**Figure 3:**
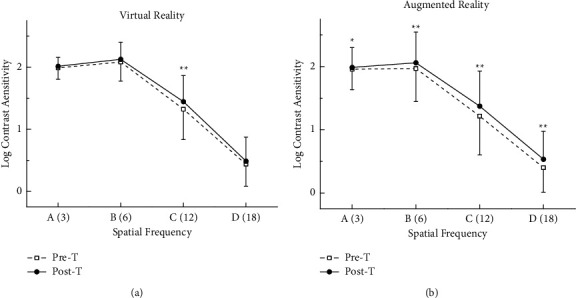
Comparison the CSF between pre- and post-training.

According to CSF, the mean log CSF of each spatial frequency improved after training both in the VR and AR groups. However, in the VR group, only the value of spatial frequency 12 improved significantly (*P* = 0.008); however, in the AR group, the value of all spatial frequencies had a statistically signiﬁcant improvement after training (*P* < 0.05). (Table 2, Figure 3).

## 5. Discussion

In amblyopia, the reduced visual acuity and the deficient monocular and binocular visual functions are the consequence of the anomalies in the visual pathway of the amblyopes, mainly in the striate and extra-striate cortex. [24] Other than conventional treatments for refractive amblyopia (patching, refractive correction by glasses or contact lens), more and more studies developed alternative treatments based on new technology over the last few year. Some researchers found that laser refractive surgery might be beneficial to improve the visual acuity significantly for the patients with refractive amblyopia [27–29]. Others found vision therapy with perceptual learning, dichoptic training, and VR-HMD seems to be an effective option for promoting visual recovery or accelerating the treatment period in amblyopia. [5, 21, 25, 26, 30–37].

In vision therapy, understanding the neural mechanisms of amblyopia is crucial for designing an effective treatment. Some studies have suggested that amblyopic eyes showed worse neural adaptation in V1, V2, V3, V3a, Vp, and V4. [38, 39] Neuroplasticity is an intrinsic property of the human brain and represents evolution's invention to enable the nervous system to escape the restrictions of its own genome and thus adapt to environmental pressures, physiologic changes, and experiences [40]. The transient reinforcement of synaptic connection contributes to short-term plasticity (STP), which quickly decays to its initial state. Moreover, the permanent change caused by repeated stimulation is in connection to the achievement of long-term potentiation (LTP) [41]. Our study found the BCVA had a significant improvement after the dichoptic short-term training based on VR and AR platform, which exhibited the short-term plasticity in refractive amblyopic children.

Stereopsis or stereoacuity is the perception of three-dimensionality because of the cortical combination between the images from each eye. To obtain correct stereoscopic perception, both eyes should have an adequate and similar visual acuity and contrast sensitivity in the presence of ocular alignment. [42] Therefore, stereopsis is one of the main features that should be assessed in the clinical management of amblyopia, since it usually is decreased or absent due to the differences in the perceived images between the amblyopic and the fellow eye. In a dichoptic binocular training, the patient perceives a scene with some parts only viewed by the amblyopic eye and some other parts by the fellow eye, while a great part of the scene is being viewed by both eyes simultaneously. In this way, the treatment focuses on stimulating not only the amblyopic eye but also on the binocularity, which is beneficial for improvement of stereoacuity [25]. Similarly, our study found the fine stereopsis was significantly improved after the dichoptic short-term training both in VR and AR groups.

Besides BCVA and fine stereopsis, CSF is also a nonnegligible clinical parameter. CSF is the ability to differentiate luminance variations between adjacent areas and offers a more complete assessment of spatial vision, which is more representative in performing everyday visual tasks, such as driving and perceiving faces [43]. It is generally believed that the CSF of amblyopic eyes declines, especially in moderate and high spatial frequencies [44]. The neural mechanism lies in alterations in the lateral geniculate nucleus and visual striate cortex [45]. We know, there is still persisting impaired CSF despite the recovery of the visual acuity after amblyopic treatment. However, several authors reported the CSF could be improved after some visual trainings [5]. Our study also found improvements in contrast sensitivity after the short-term visual training based on the VR and AR platforms. The most exciting point was the mean log CSF of AR group showed a statistically signiﬁcant improvement in all spatial frequencies. Therefore, the visual perceptual training based on AR platform seems to have a more obvious advantage in improvement on CSF than VR training. This advantage may be due to the characteristic of AR technology, which allows the user to see the real world and the computer-generated objects simultaneously, and this superimposition can supplement reality. [11, 12] Therefore, the visual perceptual training based on the AR platform may be a more efficient treatment for amblyopia.

According to our knowledge, this study was the first one to detect the vision function of amblyopic children after short-term visual training based on the VR and AR platforms simultaneously. As we hypothesized, the short-term binocular perceptual training based on VR and AR platform can significantly improve BCVA, fine stereopsis and CSF. [1, 2] However, there are some limitations that should be considered. First, this is a short-term training study, and the therapeutic effect could be fully evaluated in future with further researches of long-term training. Second, this study is a psychophysical experiment and consequently the result is associated with a certain degree of subjectivity. This fact could be solved in future with further research using imaging or neurophysiological techniques for correlating neurological findings with psychophysical experiments and their impact on amblyopia recovery [46].

## 6. Conclusion

To sum up, this preliminary clinical study demonstrated that the use of the short-term visual perceptual training based on the VR and AR technology in refractive amblyopic children can remarkably improve the visual function (including the BCVA, fine stereopsis, and CSF). Moreover, the AR training seems to be more efficient. However, future clinical trials are needed to verify if the result of short-term visual perceptual training can predict the effect of long-term visual perceptual training in amblyopia.

## Figures and Tables

**Table 1:** Participant characteristics.

	VR group	AR group	*P* value
Num. (R/L)	71 (40/31)	74 (40/34)	0.782
Age (years)	6.45 ± 2.41	7.1 ± 3.24	0.178
Sex (M/F)	34/37	44/30	0.162
**BCVA**	0.44 ± 0.17	0.43 ± 0.17	0.665

Values are means ± standard deviations for all subjects in each group.

**Table 2:** Comparison visual function between pre- and post-training.

	*VR group*			*AR group*		
	Pre-T	Post-T	P value	Pre-T	Post-T	*P* value
**BCVA**	0.44 ± 0.17	0.50 ± 0.21	**< 0.001**	0.43 ± 0.17	0.51 ± 0.21	**< 0.001**
Fine stereopsis	545.9 ± 437.3	500.0 ± 437.8	**0.018**	507.5 ± 424.7	471.6 ± 417.4	**0.029**
CSF (3, Log)	1.9 ± 0.3	2.0 ± 0.3	0.115	1.9 ± 0.4	2.0 ± 0.3	**0.038**
CSF (6, Log)	2.0 ± 0.4	2.1 ± 0.4	0.107	2.0 ± 0.5	2.1 ± 0.4	**0.002**
CSF (12, Log)	1.3 ± 0.5	1.4 ± 0.5	**0.008**	1.3 ± 0.5	1.4 ± 0.5	**0.006**
CSF (18, Log)	0.4 ± 0.3	0.5 ± 0.4	0.073	0.4 ± 0.4	0.6 ± 0.4	**< 0.001**

Values are means ± standard deviations for all subjects in each group. Pre-T: pre-training; post-T: post-training; CFS : contrast sensitivity function, bold *P* value represents <0.05. *P* value for the comparisons between VR and AR group by two-tailed paired samples *t* test.

## Data Availability

The research data used to support the findings of this study were supplied by Ethics Committee of Sichuan University under license and so cannot be made freely available. Requests for access to these data should be made to (Yongchuan Liao, yongchuan2005@163.com).
